# Effect of Sterilization and Disinfection Methods on the Physicochemical, Mechanical, and Biological Properties of Highly Porous Thermoset Polycaprolactone-Based Scaffolds

**DOI:** 10.1021/acsomega.6c00912

**Published:** 2026-03-19

**Authors:** Helin Ozsel, Anıl Ceylan, Oğul Can Erdoğan, Betül Aldemir Dikici

**Affiliations:** Izmir Institute of Technology, Department of Bioengineering, Urla, Izmir 35430, Turkey

## Abstract

Tissue engineering scaffolds intended for clinical translation must fulfill strict sterilization requirements to ensure patient safety and regulatory compliance. However, alcohol-based disinfection remains widely used in *in vitro* studies, despite failing to reflect clinically relevant terminal sterilization conditions and potentially misrepresenting scaffold performance. Here, we systematically evaluated the effects of terminal sterilization and commonly employed disinfection methods on highly porous thermoset polycaprolactone methacrylate (4PCLMA) polymerized high internal phase emulsion (PolyHIPE) scaffolds from a translational perspective. Steam autoclaving under dry, humid, and wet conditions and dry heat treatment were investigated as terminal sterilization approaches, while ultraviolet (UVC) exposure and 70% alcohol treatment were assessed as disinfection methods. Scaffold physicochemical, mechanical, and biological properties were comprehensively characterized, including thermal stability, chemical structure, pore morphology, compressive mechanical performance, sterility, and *in vitro* cell response using L929 fibroblasts. Terminal sterilization by autoclaving and dry heat preserved the chemical integrity, highly porous architecture, and compressive mechanical properties of the thermoset 4PCLMA PolyHIPE scaffolds. Crucially, only terminal sterilization methods achieved complete sterility throughout the scaffold volume, whereas UVC exposure and alcohol treatment failed to eradicate microbial contamination in these highly porous constructs. Sterilization or disinfection treatments didn't adversely affect cell viability, attachment, or proliferation. Collectively, these findings demonstrate that alcohol-based disinfection is insufficient for highly porous tissue engineering scaffolds and that 4PCLMA PolyHIPE scaffolds are compatible with clinically relevant terminal sterilization methods. This work highlights the importance of integrating appropriate sterilization strategies early in scaffold design to enhance translational relevance and clinical readiness.

## Graphical abstract

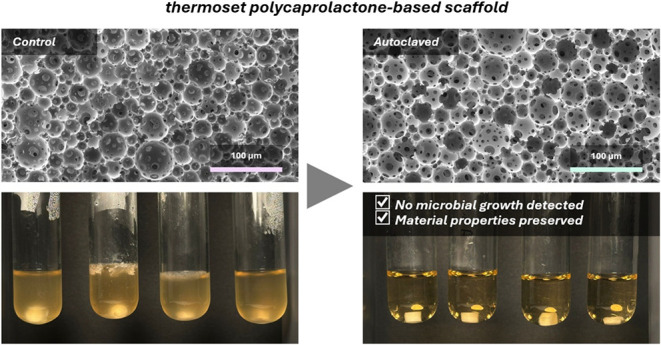

## 1 Introduction

Tissue engineering is an interdisciplinary field that applies the principles of engineering and the life sciences toward the development of biological substitutes that restore, maintain, or improve tissue function.^1^ The scaffold, serving as a temporary 3D matrix, provides structural and biochemical cues to support cell attachment, proliferation, and tissue formation. An ideal scaffold should be biocompatible, biodegradable, mechanically suitable for the target tissue, and possess a highly porous, interconnected architecture that facilitates nutrient transport and vascularization.^2^ Additionally, it must be compatible with at least one clinically acceptable sterilization method without compromising its physicochemical or biological properties to ensure safe clinical translation.^3^ In this context, clinical application requires terminal sterilization,^4^ defined as the complete elimination of all viable microorganisms, including highly resistant bacterial spores,^5^ which fundamentally differs from disinfection procedures that primarily reduce microbial load without ensuring complete sterility.

The developmental pathway for a tissue engineering scaffold from the laboratory to the clinic is a multistage process. The first critical step following fabrication is *in vitro* evaluation, during which cell-material interactions are assessed under controlled laboratory conditions. In many academic laboratories, *in vitro* cell culture experiments are routinely performed after treating scaffolds with alcohol (typically 70% ethanol) as a rapid and practical preparation step.^6,7^ However, alcohol treatment constitutes disinfection rather than sterilization, as it is effective primarily against vegetative bacteria but does not reliably eliminate bacterial spores or other resistant microorganisms. UV irradiation is also another method that is frequently used *in vitro* applications;^8–10^ however, it is also generally considered a disinfection method for solid biomaterials rather than a terminal sterilization technique.^11^ It proves to be an effective method in the sterilization of 2D surfaces in the clinic but falls short in the sterilization of 3D constructs like scaffolds due to its low penetration depth.^12^ Meanwhile, UV light around 250 nm has been reported to induce chain scission and chemical degradation in ester-containing polymers.^13^ While disinfection may be acceptable for preliminary *in vitro* screening and proof-of-concept studies, it does not reflect clinically relevant sterilization conditions.

In clinical practice, disinfection and sterilization approaches are classified according to both their antimicrobial efficacy (high-, intermediate-, and low-level) and the intended use of the medical material (critical, semicritical, and noncritical), based on the associated risk of infection.^5^ Critical materials are those that enter sterile tissues or the bloodstream and, therefore, pose a high risk of infection; examples include surgical implants, intravascular devices, and tissue engineering scaffolds intended for implantation. Semicritical materials contact mucous membranes or nonintact skin, such as endoscopes or dental instruments, while noncritical materials contact only intact skin, including stethoscopes and blood pressure cuffs.^14^ Tissue engineering scaffolds fall within the critical material category as they are implanted or directly interact with host tissues. Consequently, they must be fully sterilized prior to use to ensure patient safety and compliance with clinical and regulatory standards.^11,15,16^ Early reliance on disinfected scaffolds can, therefore, obscure sterilization-induced alterations in scaffold chemistry, morphology, or mechanical properties, potentially leading to misleading conclusions regarding biological performance and clinical readiness.

A wide range of sterilization methods has been employed for biomaterials and tissue engineering scaffolds, including heat-based techniques (steam and dry heat), radiation-based methods (gamma irradiation and electron beam), chemical sterilization (ethylene oxide), and emerging approaches such as supercritical carbon dioxide and plasma treatments. The selection of an appropriate sterilization method depends on the material’s chemical composition, thermal stability, morphology, and intended clinical application, as sterilization can significantly influence scaffold structure, mechanical properties, and biological performance.^3^ Reported effects of sterilization on scaffold properties vary widely across different material systems and fabrication strategies. Table S1 summarizes representative examples from the literature, illustrating how identical sterilization methods can lead to divergent outcomes depending on scaffold chemistry and architecture.^53–69^

Among sterilization routes, steam sterilization by autoclaving is one of the most widely used and well-established techniques in clinical and laboratory settings. Autoclaving operates based on the exposure of materials to saturated steam under elevated temperature and pressure, typically at 121–134 °C and pressures of 1–2 bar, for a defined period of time. Sterilization with an autoclave is achieved through the denaturation of proteins and nucleic acids in microorganisms, leading to the effective elimination of bacteria, viruses, fungi, and bacterial spores.^17^ Autoclaving offers several advantages, including high sterilization efficacy, reproducibility, cost-effectiveness, and the absence of toxic residues, making it the gold standard for sterilizing heat-resistant medical devices and surgical instruments. In addition, it is widely accepted by regulatory bodies and easily implemented in both clinical and industrial environments. However, its applicability is inherently limited to materials that can withstand elevated temperatures and moisture without undergoing degradation or a loss of functionality.

Steam sterilization is not a singular process but a combination of elevated temperature, pressure, and moisture exposure, each of which can independently and synergistically influence polymeric scaffold structure and performance. Previous work by Hofmann *et al.* systematically demonstrated that, even under identical autoclave temperature and pressure conditions, the presence and extent of moisture critically dictate sterilization-induced changes in porous polymeric scaffolds. By comparing dry, humid, and wet autoclaving of three-dimensional silk fibroin scaffolds, the authors showed that moisture exposure, rather than heat alone, was primarily responsible for the reduction in mechanical performance, while dry autoclaving preserved the scaffold integrity most effectively. These findings highlight moisture as a key, yet often overlooked, variable in steam sterilization of porous biomaterials.^18^

Synthetic polymers are widely used in the field of tissue engineering because of their advantages of being synthesized in a way that allows control over both chemical structures and mechanical properties of a scaffold itself, which enables high customizability of usage.^19^ Polycaprolactone (PCL) is a commercially available synthetic biodegradable polymer, which is frequently used in tissue engineering applications.^20^ Commercially available PCL is predominantly a hydroxyl-terminated thermoplastic polymer with a relatively low melting temperature (60 °C). Thermoplastic polymers soften and melt upon heating and can be reshaped repeatedly,^21^ whereas thermosets form permanently cross-linked networks (Figure 1) that do not melt upon reheating and retain their shape until degradation.^22^ As a result, widely used −OH-terminated PCL-based scaffolds are generally unsuitable for heat-based sterilization methods, such as steam autoclaving, as exposure to elevated temperatures can lead to polymer softening, deformation, loss of structural integrity, and changes in mechanical performance.^23^

**Figure 1:**
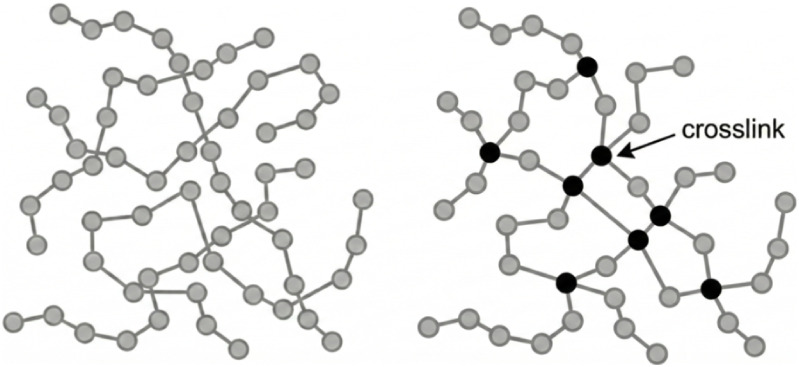
Schematic illustration of polymer chain arrangements in thermoplastic and thermoset polymers, respectively. In thermoplastic polymers, the chains are not covalently connected to each other, whereas thermoset polymers form a cross-linked network through covalent interchain connections.

As an alternative to conventional thermoplastic PCL scaffolds, acrylated^24–26^ or methacrylated PCL (PCLMA)-based scaffolds,^27–29^ representing a thermoset form of PCL, have attracted increasing interest. In our previous studies, PCLMA was employed as the structural matrix for emulsion templating, a versatile scaffold fabrication strategy based on the polymerization of high internal phase emulsions (HIPEs). In this approach, a concentrated emulsion, typically containing more than 74% (v/v) of a dispersed internal phase, is stabilized and subsequently polymerized, after which removal of the internal phase gives rise to a solid polymer network. The resulting materials, commonly referred to as polymerized HIPEs (PolyHIPEs), are characterized by a highly porous architecture composed of large, spherical, interconnected pores linked by smaller throats (windows). This intrinsic interconnectivity, combined with high porosity and tunable pore size, renders emulsion-templated PCLMA PolyHIPEs particularly attractive for scaffold applications where mass transport, cell infiltration, and three-dimensional tissue formation are critical.^30^ Using this approach, 4-arm PCLMA (4PCLMA)-based PolyHIPE scaffolds were introduced to the literature,^31^ and they have been shown to provide a supportive three-dimensional matrix for the attachment, proliferation, infiltration, and extracellular matrix (ECM) formation of bone cells,^32,33^ human fibroblasts,^31^ human mesenchymal progenitor cells,^34^ and human endothelial cells.^35^ However, in these studies, scaffolds were disinfected with 70% alcohol prior to cell culture. This approach is not only clinically irrelevant, but also the effects of alcohol treatment on the physicochemical and mechanical properties of the scaffolds have not been systematically investigated.

In this study, we address a critical translational gap in the development of emulsion-templated tissue engineering scaffolds by systematically examining the sterilization compatibility of highly porous 4PCLMA-based PolyHIPEs. Clinically relevant terminal sterilization methods, including steam autoclaving and dry heat treatment, were evaluated alongside commonly used laboratory disinfection practices such as 70% alcohol and UV irradiation, which are frequently applied in *in vitro* studies despite their limited clinical relevance. By directly comparing these approaches, this work investigates sterilization efficacy from sterilization-induced alterations in scaffold chemistry, morphology, mechanical performance, and biological response. The findings provide a framework for selecting appropriate sterilization strategies for photocurable, highly porous PCLMA scaffolds and support the alignment of routine *in vitro* testing with clinically meaningful sterilization requirements in tissue engineering.

## 2 Materials and Methods

### 2.1 Materials

Dichloromethane (DCM), diphenyl­(2,4,6-trimethylbenzoyl)­phosphine oxide/2-hydroxy-2-methylpropiophenone (photoinitiator), ε-caprolactone, methacrylic anhydride (MAAn), pentaerythritol, resazurin sodium salt, tin­(II) 2-ethylhexanoate (SnOct_2_), and triethylamine (TEA) were all purchased from Sigma-Aldrich. Dulbecco’s Modified Eagle Medium (DMEM), penicillin/streptomycin, phosphate-buffered saline (PBS), and Trypsin-EDTA were purchased from Gibco. 1,2-dichloroethane (DCE) and tryptic soy broth (TSB) were purchased from Merck. 37% hydrochloric acid (HCl), formaldehyde solution, ethanol, and methanol were purchased from Isolab. Fetal bovine serum (FBS) was purchased from Capricorn Scientific. The surfactant polyglycerol polyricinoleate (PGPR) was gifted from Palsgaard. Polydimethylsiloxane (PDMS) molds were produced using a SYLGARD Silicone Elastomer Kit. All ingredients were utilized without further purification unless otherwise indicated.

### 2.2 Synthesis and Characterization of 4PCLMA

Synthesis of 4PCLMA is explained in detail elsewhere.^33,36^ Briefly, pentaerythritol and ε-caprolactone were mixed at 160 °C in a flask until pentaerythritol was dissolved under a constant nitrogen flow. Tin­(II) 2-ethylhexanoate was added as a catalyst, and the reaction continued overnight after the pentaerythritol had completely dissolved. After dissolution, 4-arm PCL (4PCL) was cooled to room temperature. Then, 4PCL was dissolved in DCM, and TEA was added. Chemicals were stirred, and additional DCM was added to dissolve them thoroughly. The obtained mixture was transferred to an ice bath. Using a separation funnel, a solution of MAAn mixed with DCM was added to the mixture. After this process, the flask was removed from the ice bath and was stirred at 380 rpm for 68 h. The resulting 4PCLMA was sequentially washed with a 1 M HCl solution and then with deionized water. Subsequently, the solvent was removed by using a rotary evaporator. Polymer was washed with methanol and stored at −80 °C. In the following 3 days, the polymer was washed daily with methanol. When the solvent was completely removed a second time with the rotary evaporator, 4PCLMA was stored at −20 °C in glass vials. The chemical structure of 4PCLMA was confirmed by proton nuclear magnetic resonance (^1^H NMR) spectroscopy using an AVANCE III 400 MHz spectrometer (Bruker) with CDCl_3_ as solvent. Although NMR characterization has been reported previously,^33^ representative spectra are included again here to confirm the identity of the same polymer batch used in the present study.

### 2.3 Fabrication of 4PCLMA PolyHIPEs

In a glass vial, 4PCLMA (0.4 g), surfactant (PGPR) (0.06 g), DCE (1.06 g), and photoinitiator (0.06 g) were added and stirred at 370 rpm for 1 min at room temperature.^37^ Then, distilled water was added dropwise to the vial using a Pasteur pipet to the mixture while the mixture was continuously stirred for 2 min (Figure 2). HIPEs were obtained by adding 3 mL of distilled water. The emulsion was then poured into the PDMS molds and cured under the UV lamp for 1 min for each side, and PolyHIPEs were obtained (Figure 2). After curing with UV, the scaffolds were gradually transferred from 100% alcohol to 100% distilled water. This washing step was performed to remove any residual or unreacted components from the porous structure. Subsequently, the scaffolds were frozen at −20 °C and then lyophilized to remove the water while preserving the highly porous, interconnected scaffold architecture.

**Figure 2:**
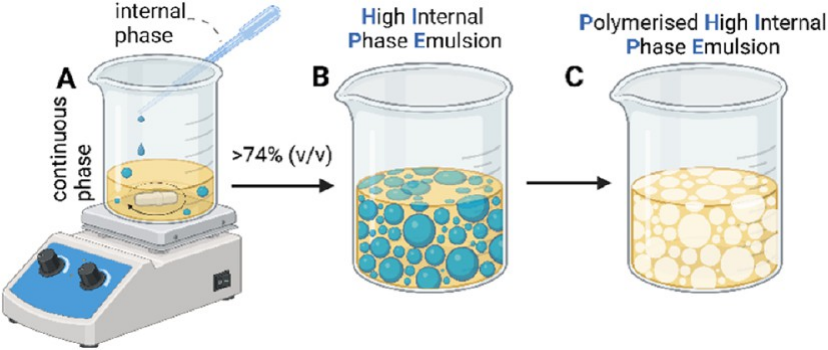
(A, B) The internal phase is added to the continuous phase while stirring. (C) After polymerization, the internal phase is removed, and the PolyHIPE scaffold is created (created using Biorender.com).

### 2.4 Thermogravimetric Analysis (TGA)

Thermal stability and degradation behavior of the PolyHIPEs were evaluated using a PerkinElmer Diamond TG/DTA Instrument. The thermogravimetric analysis (TGA) was conducted from ambient temperature up to 600 °C at a heating rate of 10 °C per minute, under a static nitrogen environment.^38^

### 2.5 Treatments and Nomenclature of Scaffolds

Following the scaffold fabrication, various sterilization and disinfection methods were applied to investigate their effects on scaffold characteristics. Scaffolds were given names depending on the way they were treated (Table 1). The control group (C_C) was stored as prepared without any additional treatment. The disinfection group (D_E) was immersed in 70% alcohol for 30 min, followed by triple rinsing with water to remove residual alcohol. For dry heat treatment (D_H), scaffolds were exposed to 180 °C in a hot-air oven for 30 min and subsequently stored in a desiccator to prevent moisture uptake. Dry autoclave treatment (A_D) was performed by placing dry scaffolds into heat-resistant bottles and sterilizing them at 121 °C for 15 min using the predefined solid-material program of a vertical steam autoclave (Nüve NC90M, Nüve, Turkey). The sterilization cycle consisted of an automated heating phase (∼15 min) until the load reached the target temperature, followed by a 15 min holding phase at 121 °C and a controlled cooling phase (∼20 min) prior to cycle completion. During sterilization, a saturated steam environment corresponding to approximately 1.2 bar gauge pressure was automatically generated by the instrument. Pressure regulation, temperature ramping, and cooling were automatically controlled according to the manufacturer-defined operating conditions and were not manually adjusted. For humid autoclave treatment (A_H), scaffolds were immersed in distilled water, excess water was removed using filter paper, and then they were autoclaved under the same conditions. In the wet autoclave group (A_W), scaffolds were fully immersed in deionized water inside heat-resistant bottles and autoclaved using the same autoclave cycle conditions. Finally, the Ultraviolet (UVC) treatment group (U_V) was exposed to UVC radiation for 1 h.^39^ All scaffold groups were lyophilized after treatment.

**Table 1:** Experimental Groups and Their Sterilisation/Disinfection Conditions

Group name	Condition	Details
C_C	Control group	-
A_D	Dry autoclaved	121 °C, 15 min
A_H	Humid autoclaved	121 °C, 15 min
A_W	Wet autoclaved	121 °C, 15 min
D_H	Dry heat	180 °C, 30 min
U_V	Ultraviolet treatment	1 h, 254 nm UVC
D_E	Alcohol treatment	70% alcohol, 30 min

### 2.6 Fourier Transform Infrared (FTIR) Spectroscopy

To evaluate the impact of treatments on the chemical structure of PolyHIPE scaffolds in detail, we conducted Fourier Transform Infrared (FTIR) spectroscopy analysis. Samples were analyzed using an FTIR spectrometer equipped with an Attenuated Total Reflectance (ATR) accessory, with air taken as a background. The spectra were recorded in the range of 4000 to 400 cm^–1^. The absorption bands identified in the FTIR spectra correspond to the functional groups present in the scaffolds’ chemical structure. This method was used to detect possible chemical alterations due to the conditions.

### 2.7 X-ray Diffraction (XRD)

X-ray diffraction (XRD) patterns of PolyHIPEs were recorded to investigate the crystalline structure of sterilized and disinfected samples using a diffractometer (Rigaku Miniflex, Japan).^40^ Cu–Kα radiation was used, and freeze-dried samples were scanned at 5°/min in the angular range (2θ) from 5° to 60°.

### 2.8 Morphological Characterization

The microstructural evaluation of the scaffolds was performed by using scanning electron microscopy (SEM). Cross sections were obtained using a surgical scalpel and mounted on aluminum stubs with carbon adhesive. The samples were sputter-coated with gold at 15 kV for 2.5 min to enhance conductivity before imaging. 100 pore and 100 window diameters were measured per group using ImageJ software. The obtained pore diameter measurements were then adjusted using a statistical correction factor of 2/√3,^41^ and graphs showing the distribution of both pore and window sizes were subsequently prepared.

### 2.9 Mechanical Characterization

The mechanical properties of the 4PCLMA-based scaffolds were evaluated by using a compact benchtop universal testing machine equipped with a 100 N load cell (EZ-TEST EZ-S, Shimadzu, Japan). Cylindrical specimens with an approximate diameter of 3.9 mm and a height of 3.5 mm were prepared for compression testing. Following the respective sterilization or disinfection treatments, all scaffold samples were freeze-dried to ensure consistent testing conditions and to remove residual moisture prior to mechanical evaluation. The exact dimensions of each sample were measured and recorded by using a digital caliper. The tests were performed at a constant rate of 1 mm/min until approximately 70% strain was reached. The compressive modulus was determined from the slope of the initial linear region of the resulting stress–strain curve for each sample.^42^

### 2.10 Test for Sterility

The sterility of all scaffolds was assessed using a growth-based immersion culture approach adapted from European Pharmacopoeia sterility testing principles^43^ and conceptually consistent with sterility testing strategies described in ISO 11737-2. The assessment was designed to evaluate the elimination of intrinsic bioburden accumulated during the nonaseptic fabrication and handling processes rather than to perform formal sterilization validation using predefined microbial challenges. The potential contaminants, therefore, included naturally occurring environmental microorganisms, such as bacteria and fungi, introduced during routine laboratory processing.

TSB was used for general microbial detection, while antibiotic-free DMEM supplemented with 10% FBS was employed to evaluate sterility under cell culture-relevant conditions. In a sterile environment, scaffolds were placed in T-25 flasks and fully immersed in the respective media. Samples were briefly vacuum-treated to promote complete infiltration of the culture medium into the interconnected pore network and to facilitate the recovery of microorganisms potentially residing within internal scaffold regions.

The samples were incubated at 37 °C for 21 days in DMEM and 5 days in TSB to allow the detection of slow-growing microorganisms. Throughout the incubation period, flasks were visually inspected and documented for signs of contamination, indicated by turbidity in TSB or a phenol red color change from red to yellow in DMEM resulting from microbial metabolic activity. No biological indicator organisms (e.g., *Geobacillus stearothermophilus*) were intentionally inoculated; therefore, the results should be interpreted as a comparative evaluation of microbial elimination efficiency between treatments rather than a determination of sterility assurance level (SAL).

### 2.11 Biological Characterization

#### 2.11.1 Cell Culture

To evaluate the metabolic activity and proliferation behavior of cells on the scaffolds following various treatments, L929 fibroblast cells were used. Cells were retrieved from liquid nitrogen and thawed into a T-75 culture flask containing DMEM supplemented with 10% FBS, 2 mM l-glutamine, and 100 mg/mL penicillin/streptomycin. Cells were incubated at 37 °C in a humidified atmosphere with 5% CO_2_. Cultures were maintained until they reached approximately 90% confluency, and the medium was refreshed every 2–3 days.

#### 2.11.2 Cell Seeding

Cylindrical scaffolds with the same dimensions used for mechanical testing (approximately 3.9 mm in diameter and 3.5 mm in height) were transferred aseptically into 48-well culture plates, with one scaffold per well. 5 μL of cell suspension with a concentration of 12 × 10^3^ was homogeneously pipetted and inoculated onto each scaffold. To allow proper cell attachment, scaffolds were incubated for 45 min at 37 °C and 5% CO_2_ without adding additional medium. To prevent scaffold dehydration and maintain humidity, sterile deionized water was added to the spaces between the wells. Medium was added, and scaffolds were maintained in culture for 5 days, while the medium was changed every 2–3 days.^33^

#### 2.11.3 Metabolic Activity

To assess and compare the metabolic activity of the cells, we performed a Resazurin reduction (RR) assay. A 1 mM stock solution of resazurin (*M*_w_: 251.17 g/mol) was prepared, sterile-filtered, and diluted to 100 μM in culture medium under aseptic conditions. 750 μL of the resazurin working solution was added to each well in a 48-well plate, and scaffolds were transferred into the wells using sterile forceps. The culture plate was protected from light and incubated for 4 h at 37 °C in a CO_2_ incubator. Following incubation, 200 μL of the reduced solution was transferred from each well to a 96-well plate in triplicate, and relative fluorescence was measured using a spectrofluorometer at 540 nm excitation and 635 nm emission wavelengths.^35^ After the assay, scaffolds were washed twice with PBS, and fresh medium was added to allow for continued incubation for further analysis. The RR assay was performed at three different time points (days 1, 3, and 5) using at least three fresh scaffolds per time point.

#### 2.11.4 Biological SEM

For SEM characterization, 4PCLMA PolyHIPE scaffolds were first submerged in PBS and subsequently washed with deionized water for 5 min. To achieve dehydration, samples were subjected to a graded alcohol series (35%, 60%, 80%, 90%, and 100%), each for 15 min. Following dehydration, the scaffolds were incubated in a 1:1 solution of hexamethyldisilazane (HMDS) and alcohol for 1 h and then treated with pure HMDS for 5 min. The samples were then left to air-dry overnight in a laminar flow cabinet. The dried scaffolds were subsequently sputter-coated with a conductive layer and imaged using SEM to assess their surface morphology and porous microarchitecture. False-color rendering was applied to enhance the visual interpretation of cell morphology using Adobe Photoshop. Quantitative analyses of adhesion-related markers or cytoskeletal organization were not performed within the scope of this study.

### 2.12 Statistical Analysis

Statistical analyses were performed using GraphPad Prism 9 software. Data were reported as mean ± standard deviation (SD), and samples were analyzed with one-way analysis of variance (ANOVA) for the mechanical test and two-way ANOVA for metabolic activity. A *p*-value of less than 0.05 was considered statistically significant. All experiments were conducted with at least three replicates per group.

## 3 Results and Discussion

### 3.1 Synthesis of 4PCLMA and Development of 4PCLMA PolyHIPEs

The previously synthesized 4PCLMA prepolymer was characterized by ^1^H NMR spectroscopy to confirm its chemical structure (Figure 3). The spectrum exhibited characteristic methacrylate signals, including vinyl proton peaks at 5.5 and 6.1 ppm and a methyl proton peak at approximately 1.9 ppm. The absence of the hydroxyl-associated signal previously observed at around 3.6 ppm indicates almost complete methacrylation of the polymer. Although the NMR characterization of this material has been reported previously,^33^ the spectrum is presented here again to verify the identity of the same polymer batch used in this study. High methacrylation efficiency was targeted to maximize cross-link density and mechanical integrity within the resulting scaffold networks.

**Figure 3:**
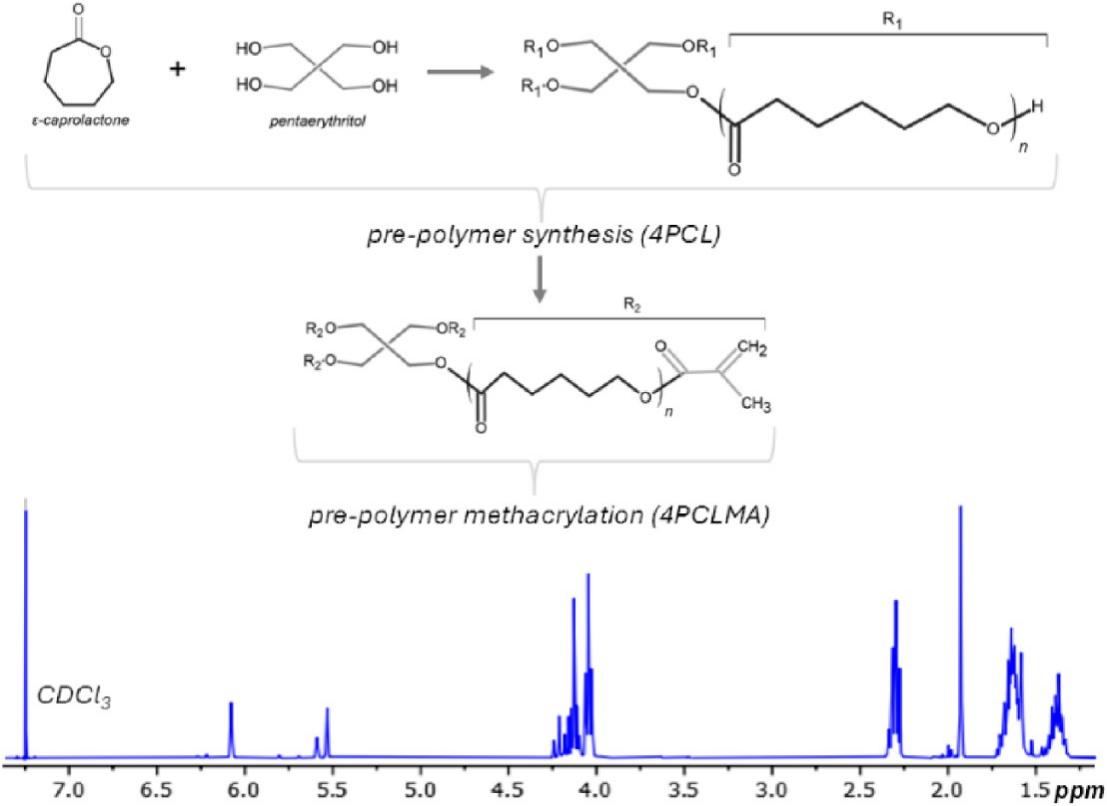
(Top) Synthesis of 4PCLMA: 4-armed PCL synthesis from the ε-caprolactone monomers and pentaerythritol; methacrylation of hydroxyl end groups. Representative ^1^H NMR spectrum of 4PCLMA confirms successful methacrylation. Detailed synthesis and characterization of this polymer have been reported previously;^33^ the spectrum is shown here to confirm the identity of the same polymer batch used in the present study.

Following prepolymer synthesis, emulsions with an internal phase volume of 82% were successfully prepared. The resulting emulsions remained stable throughout processing, exhibiting no observable phase separation, and were readily cast into PDMS molds to yield fully cured scaffolds following light-induced cross-linking. SEM revealed a highly porous PolyHIPE architecture characterized by a homogeneous distribution of spherical pores interconnected by well-defined windows across multiple length scales (Figure 4). Quantitative image analysis indicated an average pore diameter of 45.9 ± 20.7 μm and a mean window size of 8.8 ± 3.9 μm, corresponding to a d/D ratio of 0.19, which is indicative of effective pore interconnectivity. The combination of large pores and interconnected windows is considered favorable for mass transport and cell infiltration in tissue engineering applications.^37^

**Figure 4:**
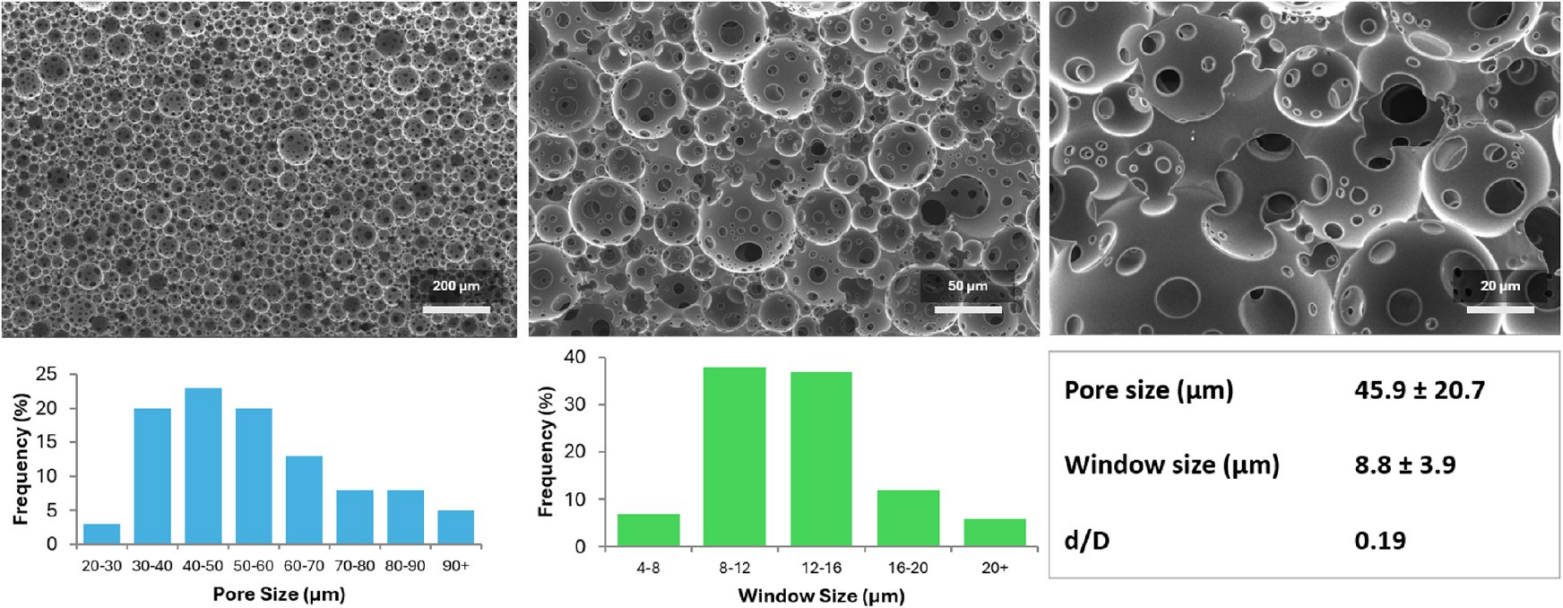
(Top) SEM images, (bottom) pore size distribution, and window size distribution of 4PCLMA PolyHIPEs.

While the average pore size obtained in this study is slightly below the commonly cited minimal range for bone tissue (50–100 μm),^44^ the relatively broad pore size distribution indicates the presence of pores exceeding this threshold. Moreover, previous studies have demonstrated that smaller pores combined with interconnected windows can promote early-stage cell attachment and migration by increasing the surface roughness and enhancing cell-material interactions. For instance, Bohner and Baumgart reported that such architectures can facilitate early cellular responses despite reduced pore diameters.^45^ Similarly, Owen *et al.* demonstrated that PolyHIPE scaffolds with intrinsic pore sizes in the range of 20–30 μm, fabricated by varying the ratios of 2-ethylhexyl acrylate and isobornyl acrylate, supported favorable cell-material interactions when combined with macroporosity.^46^ Furthermore, the emulsion templating approach offers substantial flexibility in tailoring pore architecture, allowing pore size and interconnectivity to be readily adjusted through compositional or processing modifications once structural stability under sterilization conditions has been established.^37^

### 3.2 TGA Analysis

The thermal stability of 4PCLMA PolyHIPE scaffolds was evaluated by using TGA, as shown in Figure 5. The thermogram indicates that the material remains thermally stable up to approximately 300 °C, with the onset of significant mass loss occurring at higher temperatures. The corresponding derivative weight curve (Figure 5B) reveals a major degradation event centered around 400 °C, confirming that the primary thermal decomposition of the polymer network occurs well above the temperatures applied during sterilization. Notably, no measurable mass loss was observed at 180 °C, demonstrating that the scaffolds retain their thermal integrity under the most severe sterilization condition investigated in this study (D_H). The observed thermal stability is attributed to the thermoset nature of the 4PCLMA network. In contrast to thermoplastic PCL, which may undergo thermal softening and structural deformation when exposed to elevated temperatures, cross-linked polymer networks exhibit restricted chain mobility and enhanced resistance to thermal stress. This distinction is consistent with previous reports evaluating sterilization effects on biodegradable polymeric scaffolds, where thermoplastic materials were shown to be more susceptible to thermal deformation during sterilization processes, even in the absence of significant mass loss. Such behavior has been reported for PCL-based systems subjected to elevated temperatures, where changes in mechanical integrity and structural stability were observed despite limited chemical degradation.^47^ Overall, our findings demonstrate that the thermoset architecture of 4PCLMA PolyHIPE scaffolds provides sufficient thermal robustness to withstand high temperatures without compromising structural integrity, supporting their suitability for applications requiring clinically relevant sterilization protocols.

**Figure 5:**
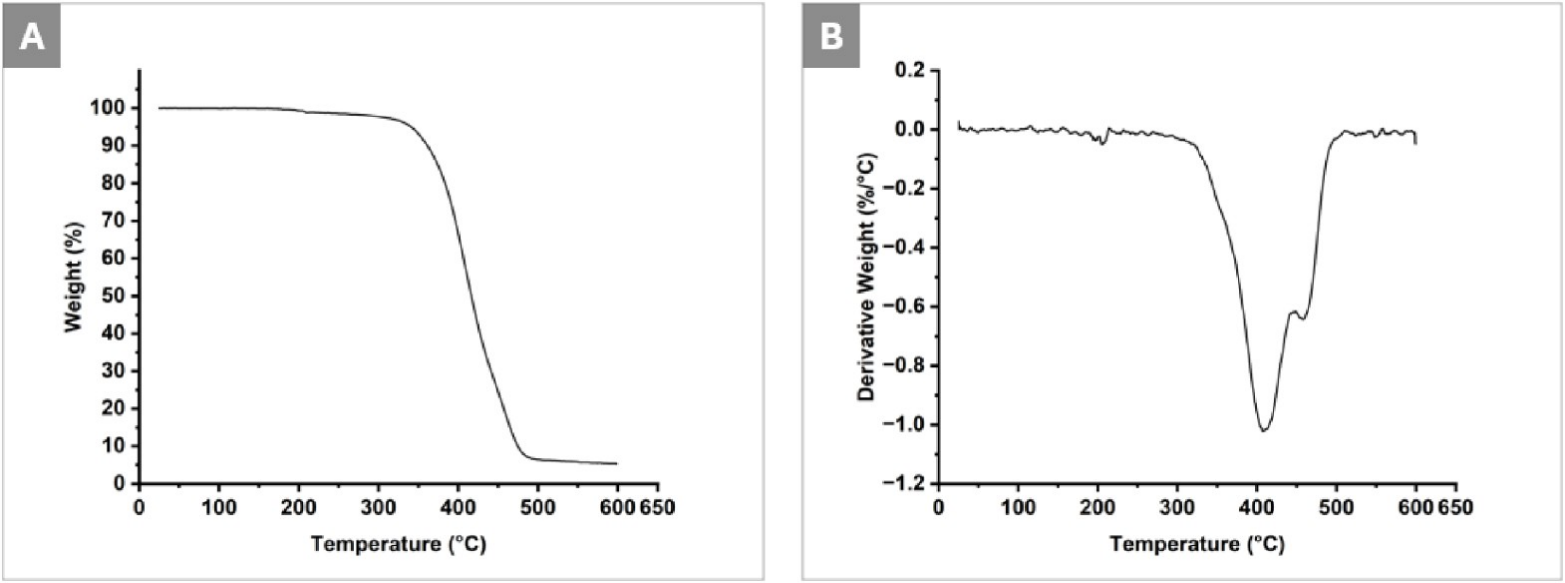
TGA analysis of PolyHIPE scaffolds (A) weight loss versus temperature and (B) derivative weight loss versus temperature.

### 3.3 FTIR Spectroscopy

FTIR spectroscopy was employed to assess whether the applied sterilization and disinfection procedures induced chemical alterations in the PolyHIPE scaffolds (Figure 6). The spectra revealed characteristic absorption bands corresponding to the functional groups of the polymer network. In particular, the O–H stretching region (3200–3600 cm^–1^), which may be indicative of hydrolytic processes, showed no significant changes following any of the applied treatments. Similarly, the CH_2_ stretching vibrations in the 2800–3000 cm^–1^ range remained consistent across all samples, suggesting the absence of chemical degradation detectable by FTIR. Furthermore, the characteristic ester carbonyl (C=O) stretching band between 1700 and 1750 cm^–1^ and the C–O stretching vibrations in the 100–1200 cm^–1^ region exhibited no peak shifts and only minor intensity variations, which were not indicative of chemical degradation. These observations indicate that the chemical structure of the PolyHIPE scaffolds remained stable within the detection limits of FTIR analysis.

**Figure 6:**
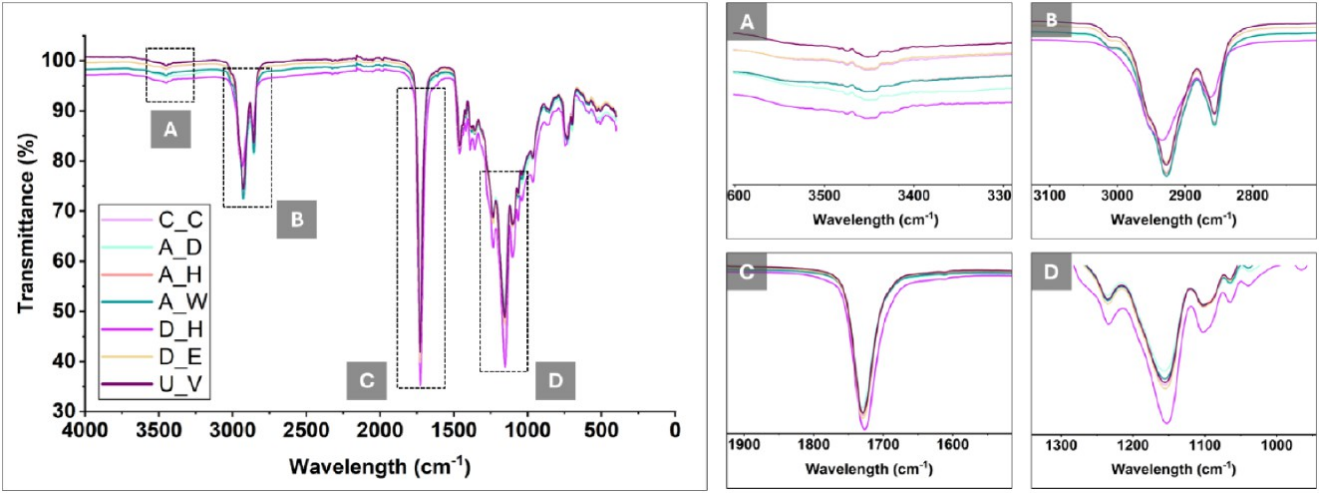
FTIR spectra of treated 4PCLMA PolyHIPEs compared to the control.

Comparable observations were reported by Griffin *et al.*, who investigated the effects of various sterilization techniques on POSS/PCL and POSS/polycarbonate urethane (PCU) nanocomposite scaffolds.^47^ In their study, FTIR analysis revealed no significant alterations in the characteristic functional groups following autoclaving, UV irradiation, gamma irradiation, or plasma treatment, indicating preservation of the polymer chemical structure within the detection limits of the technique. Importantly, while thermoplastic polymers such as PCL may undergo thermal softening or structural deformation upon exposure to elevated temperatures, these effects are not necessarily associated with chemical degradation detectable by FTIR analysis. In contrast, the cross-linked architecture of thermoset systems restricts polymer chain mobility and provides enhanced resistance to thermally induced deformation.

In line with these findings, the present results indicate that the applied sterilization and disinfection procedures did not induce detectable chemical degradation, such as ester bond cleavage, in the PolyHIPE scaffolds. The preservation of characteristic functional groups across all treatment conditions supports the chemical stability of the 4PCLMA-based matrices.

### 3.4 XRD

XRD is widely employed to evaluate the crystalline structure and phase composition of materials.^48^ The XRD patterns of all 4PCLMA PolyHIPE scaffolds exhibited a broad, low-intensity diffraction halo, with no sharp crystalline reflections detected across the measured range, indicating a predominantly amorphous polymer structure (Figure S1). In contrast, high-molecular-weight, hydroxyl-terminated linear PCL is well-known to exhibit a semicrystalline structure characterized by distinct diffraction peaks at 2θ ≈ 21.3° and 23.6°, corresponding to the (110) and (200) planes of the orthorhombic crystalline phase.^49^ No such crystalline reflections were observed for the 4PCLMA PolyHIPE scaffolds in this study. This deviation from conventional PCL behavior can be attributed to the low molecular weight of the PCL prepolymer and the formation of a cross-linked thermoset network, both of which restrict regular chain packing and inhibit crystallite formation. In line with this interpretation, Utroša *et al.* reported that increasing cross-linking density disrupts PCL chain organization, resulting in reduced crystalline order in cross-linked PCL PolyHIPE foams.^50^

The diffraction profiles of the sterilized and disinfected scaffolds closely matched those of the untreated control, with no observable shifts in peak position or emergence of crystalline reflections following treatment. The amorphous diffraction behavior was therefore preserved. These findings indicate that the applied sterilization and disinfection procedures did not induce crystallization or alter the phase structure of the 4PCLMA PolyHIPE scaffolds within the detection limits of XRD analysis.

### 3.5 Morphological Characterization

Macroscopic inspection of the 4PCLMA PolyHIPE scaffolds showed no visible changes in shape or overall morphology following sterilization and disinfection treatments (Figure 7). All samples retained their original geometry and macroscopic dimensions, with no evidence of deformation, shrinkage, or cracking, except for a slight yellowish coloration observed in the dry heat (D_H) group.

**Figure 7:**
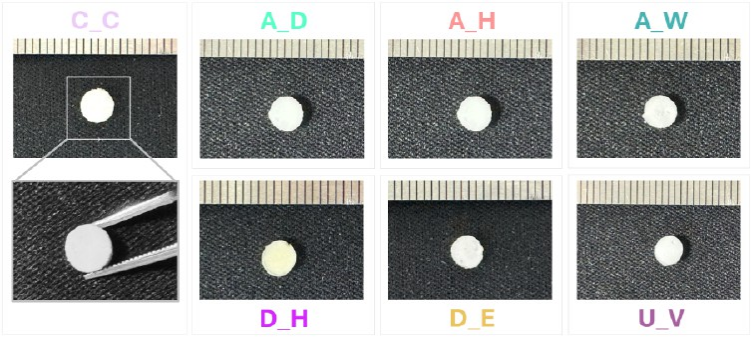
Representative macroscopic images of the scaffolds before (C_C) and after treatments. The ruler shown in the images has 1 mm spacing between adjacent markings.

To further investigate whether these macroscopic observations were reflected at the microscale, the scaffold morphology was subsequently examined using SEM. As shown in Figure 8, and also discussed in Section 3.1, the untreated control group (C_C) exhibited a highly porous and homogeneous PolyHIPE architecture characterized by well-defined, spherical pores and interconnected windows. Comparable microstructural features were observed across all treated groups, with no discernible differences in pore morphology, size distribution, or overall structural organization following sterilization or disinfection. No evidence of pore collapse, melting, or structural damage was detected, even under the most thermally demanding treatment conditions. These findings demonstrate that the applied sterilization protocols preserved the microscale architecture of the 4PCLMA PolyHIPE scaffolds, consistent with the macroscopic observations and supporting their structural robustness under clinically relevant sterilization conditions.

**Figure 8:**
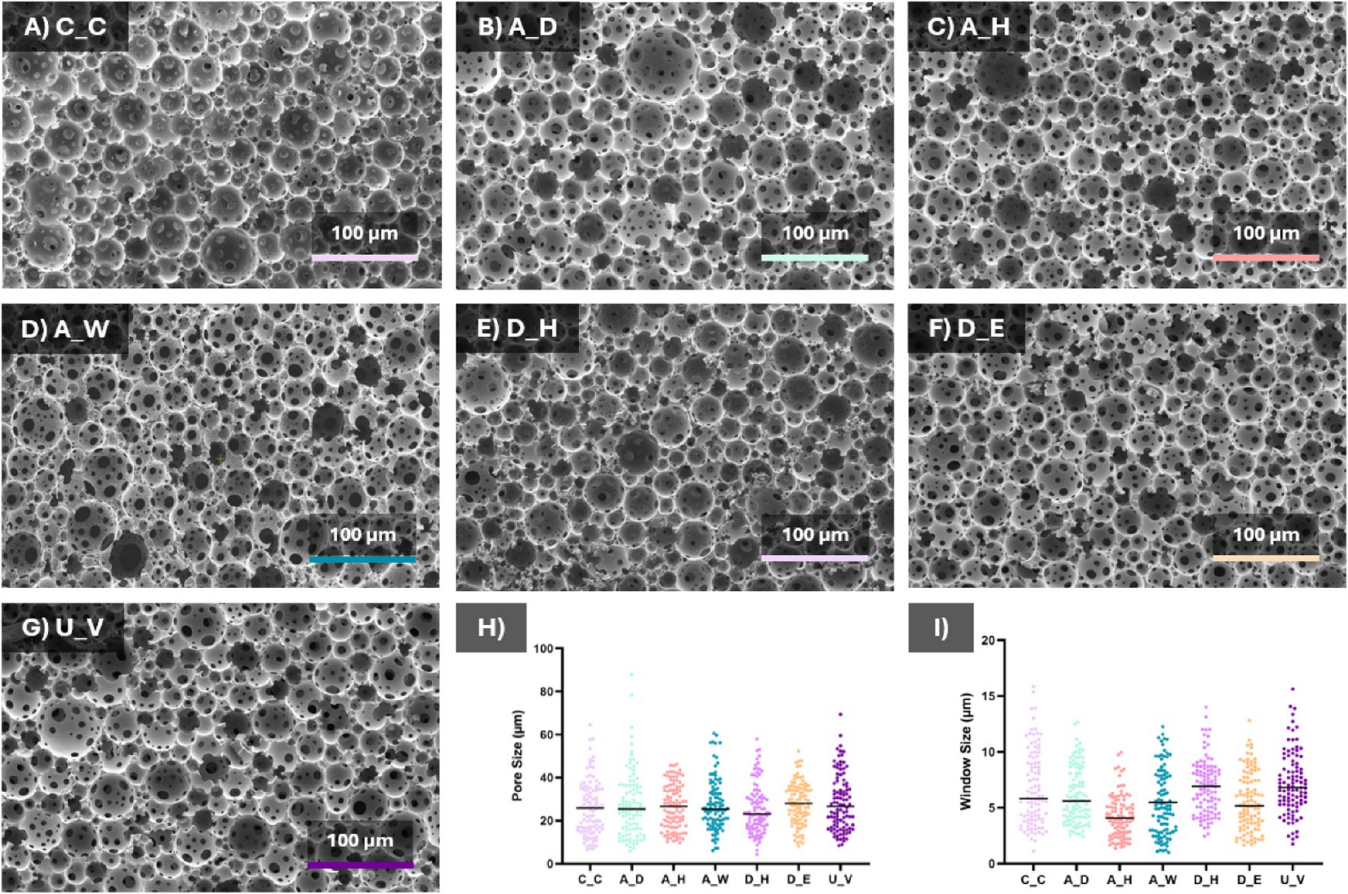
(A–G) SEM images of scaffolds after treatments compared to the control. (H) Pore size and (I) window size of distributions of the scaffolds.

In contrast, Yoganarasimha *et al.* reported pronounced melting and fiber fusion in electrospun PCL scaffolds following autoclave sterilization.^51^ It is important to note that their study did not involve methacrylated or multiarm PCL systems. Differences in polymer chemistry, molecular architecture, and the absence of a covalently cross-linked network likely contributed to the reduced thermal stability observed. Moreover, variations in scaffold fabrication strategy, particularly between electrospinning and emulsion templating, may further explain the divergent structural responses to thermal exposure.

Consistent with these qualitative observations, quantitative image analysis in Figure 8 revealed no statistically significant differences in average pore size or window size among the C_C, A_D, A_H, A_W, D_H, D_E, and U_V groups. Although minor variations in pore and window size distributions were detected, these differences were not statistically significant. Collectively, these results demonstrate that the applied sterilization and disinfection protocols effectively preserved both the microstructural integrity and pore architecture of the 4PCLMA PolyHIPE scaffolds.

### 3.6 Mechanical Characterization

The compressive mechanical behavior of the PolyHIPE scaffolds was evaluated using uniaxial compression testing, and representative stress–strain curves are shown in Figure 9A. All scaffold groups exhibited a characteristic nonlinear mechanical response, consisting of an initial elastic region followed by progressive deformation at higher strain levels, which is typical for highly porous polymeric architectures. Quantitative analysis of the compressive modulus, calculated from the linear region of the stress–strain curves, is presented in Figure 9B. The mechanical testing of the present 4PCLMA PolyHIPE scaffolds revealed stiffness values ranging from approximately 0.05 to 0.10 MPa (Figure 9B). The measured compressive modulus values are substantially lower than those of native trabecular bone, which is expected given the highly porous architecture of the PolyHIPE scaffolds. The reported values represent the bulk mechanical response of the scaffold rather than the intrinsic stiffness of the polymer phase with which cells locally interact. Accordingly, these materials are not intended for load-bearing applications but are designed to function as temporary matrices supporting tissue infiltration and regeneration.

**Figure 9:**
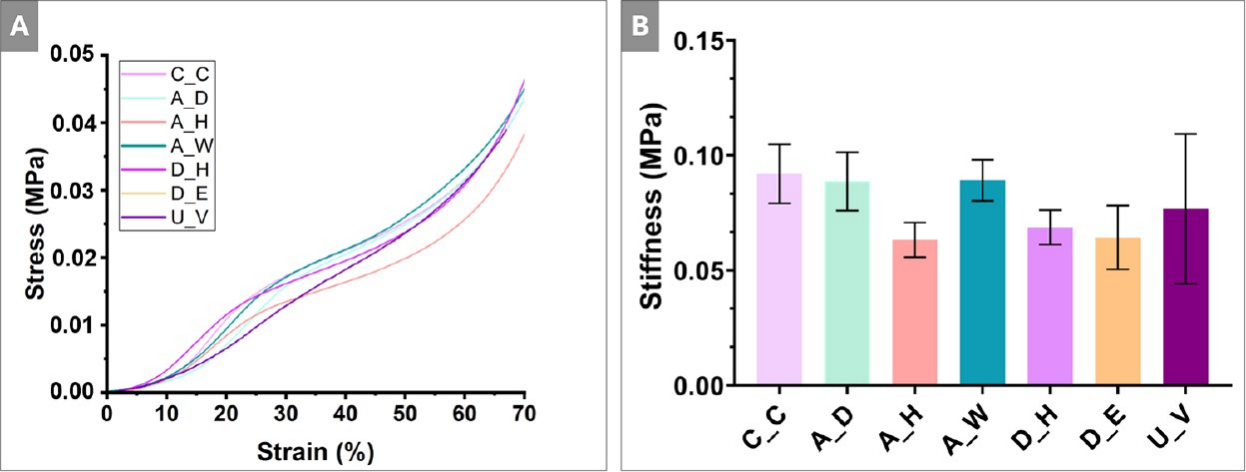
(A) Stress–strain curve and (B) stiffness of the 4PCLMA PolyHIPE scaffolds. Statistical analysis was performed using one-way ANOVA, and no significant differences were observed between the experimental groups and the control (*p* > 0.05) (*n* = 4).

Overall, the majority of sterilization and disinfection treatments did not result in significant changes in scaffold stiffness when compared to the untreated control (C_C). While the A_H and D_E groups exhibited a slight reduction in the compressive modulus, these differences were not statistically significant. These findings indicate that the applied sterilization and disinfection protocols did not compromise the mechanical integrity of the PolyHIPE scaffolds. In agreement with this observation, Böhm *et al.* reported that medical-grade PCL retains its mechanical performance, including elastic modulus and crystallinity, when exposed to moderate thermal conditions, despite minor morphology-related changes.^52^ Extending these findings, the present study demonstrates that even exposure to higher temperatures associated with sterilization (up to 180 °C) does not adversely affect the compressive mechanical performance of cross-linked 4PCLMA PolyHIPE scaffolds.

Mechanical testing in this study was conducted under dry conditions following freeze-drying to enable consistent comparison of structural integrity after different sterilization and disinfection treatments. While this allows direct assessment of sterilization-induced structural changes, mechanical behavior under physiological conditions may differ due to hydration-dependent polymer chain mobility and network relaxation.^32^ Water uptake can act as a plasticizing agent, potentially reducing stiffness in highly porous polymeric scaffolds. Therefore, although structural integrity was preserved following sterilization, mechanical performance under hydrated conditions may vary, and future studies evaluating behavior after equilibration in aqueous media will further support translational assessment.

### 3.7 Test for Sterility

The efficacy of the applied sterilization and disinfection methods was evaluated using a sterility test based on incubation in DMEM for 21 days and TSB for 5 days (Figure 10). In DMEM, visible contamination, indicated by a color change of the phenol red indicator from red to yellow, was observed only in the untreated control group (C_C), while all treated samples remained visually clear throughout the incubation period (Figure 10). This observation is consistent with previous reports showing that DMEM can be used as an initial indicator of contamination due to pH shifts associated with microbial metabolism. However, pH-based indicators may fail to detect early or low-level contamination, particularly when microbial metabolic activity does not produce sufficient acidic byproducts to induce a visible color change.

**Figure 10:**
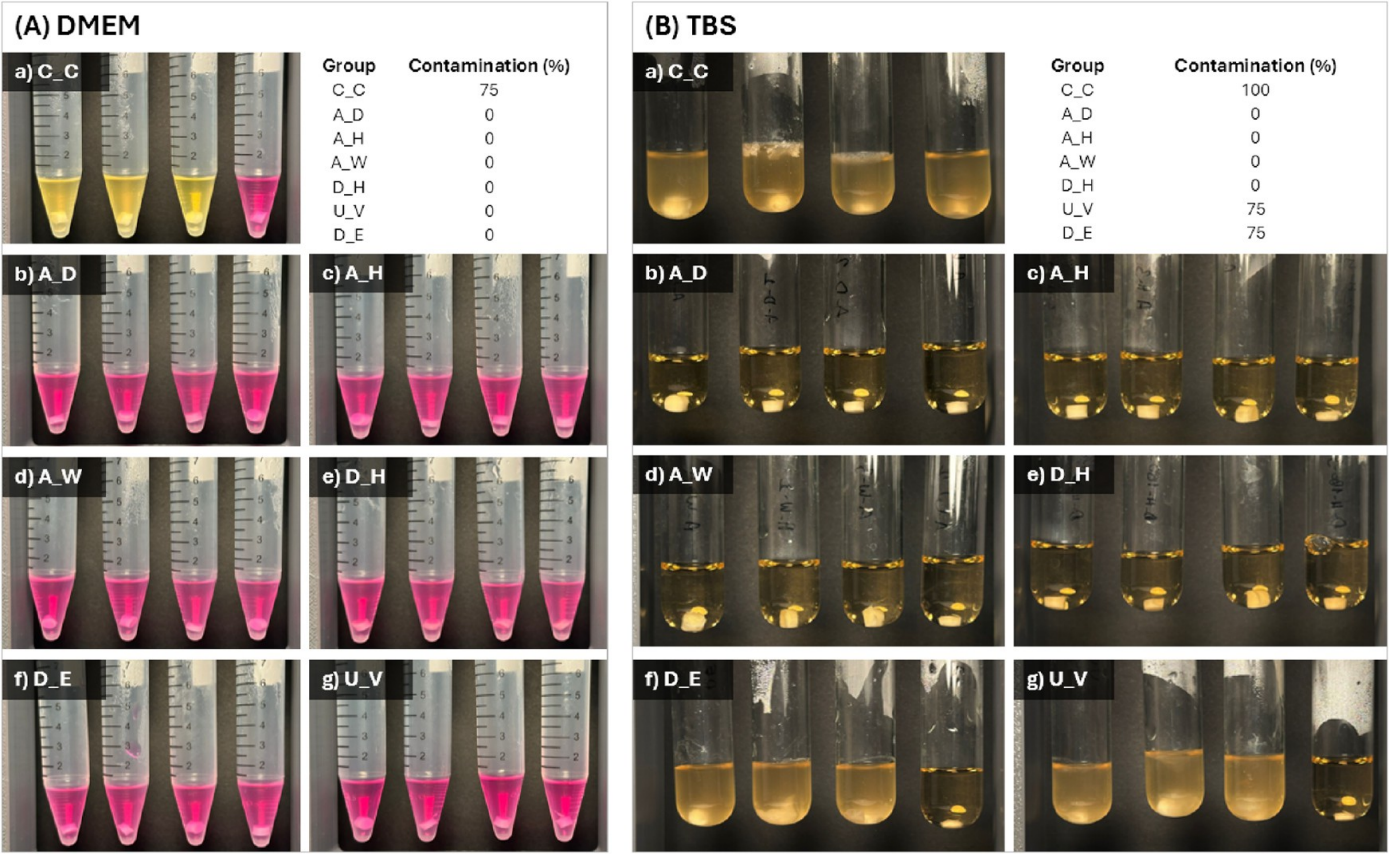
Sterility test results after 21 and 5 days of incubation in DMEM and TSB, respectively (*n* = 4).

In contrast, incubation in Tryptic Soy Broth, a nutritionally rich medium recommended for sterility testing by the European Pharmacopoeia, provided a more sensitive assessment of microbial contamination.^43^ Under these conditions, turbidity was observed in the untreated control (C_C) as well as in the U_V and D_E groups, whereas all samples subjected to terminal sterilization methods remained clear (Figure 10). These results indicate that, while UV irradiation and alcohol treatment may reduce surface microbial load, they are insufficient to ensure absolute volumetric sterility within these highly porous scaffolds.

Importantly, the porous architecture of the PolyHIPE scaffolds likely contributes to this behavior. While alcohol treatment was performed using a gradual wetting protocol to maximize penetration, complete infiltration of highly interconnected pore networks cannot be guaranteed. Similarly, the limited penetration depth of UV irradiation restricts its effectiveness in sterilizing three-dimensional porous structures. In contrast, terminal sterilization methods such as autoclaving and dry heat effectively eliminated microbial contamination, demonstrating their suitability for ensuring sterility in highly porous polymeric scaffolds.

When interpreting these results, it is important to distinguish between surface disinfection and volumetric sterility. While UVC exposure and alcohol treatment may reduce microbial contamination at exposed scaffold surfaces, the interconnected porous architecture can limit penetration of these disinfection approaches, allowing microorganisms residing within internal regions to survive. The growth-based sterility assessment used in this study, therefore, evaluates the presence or absence of viable contamination throughout the scaffold volume rather than quantitative microbial reduction. Although quantitative microbiological methods such as colony-forming unit (CFU) enumeration could provide additional information about microbial load reduction, the present findings demonstrate that the tested disinfection approaches were insufficient to achieve complete sterility within the porous scaffold structure.

Collectively, these results demonstrate that while surface disinfection methods may be sufficient for nonporous materials, they are inadequate for ensuring the volumetric sterility of porous PolyHIPE scaffolds. Terminal sterilization approaches are therefore essential to achieve reliable sterility and ensure safe application of these materials in biologically relevant environments.

### 3.8 Biological Characterization

Metabolic activity analysis demonstrated that L929 cells remained viable on all scaffolds throughout the culture period, indicating overall cytocompatibility following both sterilization and disinfection treatments (Figure 11A). An increase in metabolic activity over time was generally observed, consistent with progressive cell adaptation and proliferation within the porous scaffold architecture. While variations in metabolic response were detected between treatment groups at individual time points, most differences relative to the D_E reference condition were not statistically significant, suggesting that the applied sterilization methods did not adversely affect cellular compatibility. The higher variability observed in certain groups may reflect differences in early cell–material interactions, local cell distribution within the interconnected pore network, and assay sensitivity to metabolic state rather than direct cytotoxic effects. Overall, the results indicate that sterilization treatments preserved the ability of the scaffolds to support cell attachment and metabolic activity.

**Figure 11:**
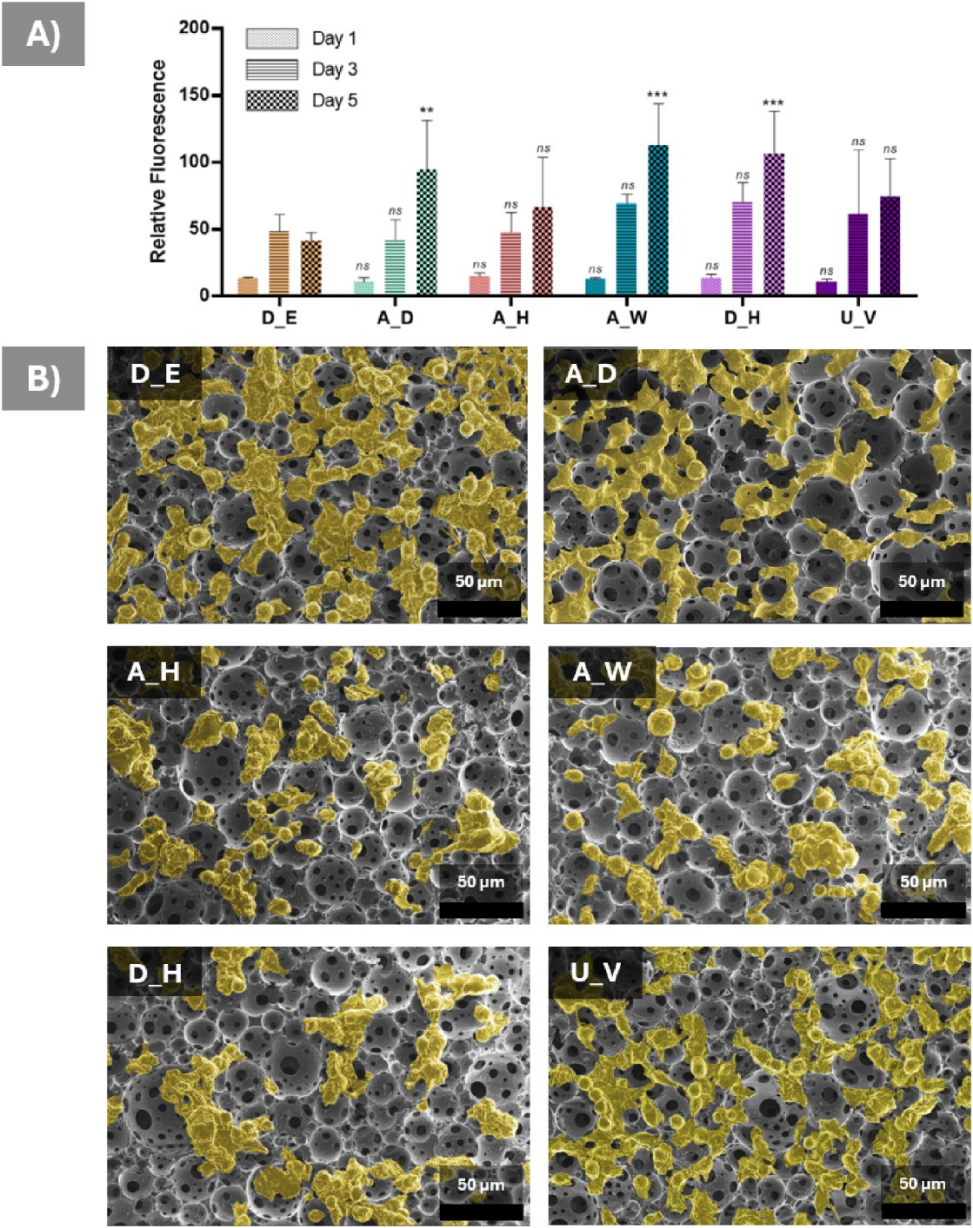
Metabolic activity of cells and morphological characterization. (A) Resazurin reduction assay results of L929 cells on days 1, 3, and 5 (*n* = 4). Statistical analysis was performed using two-way ANOVA. For each time point, treatment groups were compared against the corresponding D_E group. Statistical significance is indicated as follows: **: *p* < 0.01, ***: *p* < 0.005, ns: not significant, *p* > 0.05. (B) Representative false-colored biological SEM images showing L929 cell attachment and distribution on treated 4PCLMA PolyHIPE scaffolds.

SEM further supported the cytocompatibility findings by revealing successful cell attachment and spreading across all treatment groups (Figure 11B). L929 cells were observed to adhere to the scaffold surfaces and bridge across pore walls, demonstrating active interaction with the interconnected porous architecture. False-color enhancement highlights the widespread distribution of cells throughout the scaffold surface, indicating that sterilization and disinfection treatments did not impair initial cell adhesion behavior. Representative uncolored SEM images are provided in the Supporting Information (Figure S2) to allow direct visualization of the original micrographs. While minor differences in local cell density were visible between samples, such variations are commonly associated with heterogeneous cell seeding and the complex topology of highly porous scaffolds rather than treatment-induced effects. Overall, the SEM observations corroborate the metabolic activity results, confirming preservation of cell-material compatibility following sterilization. The biological response, therefore, reflects both metabolic activity and qualitative morphological evidence of cell attachment and spreading rather than quantitative analysis of adhesion-related markers.

## 4 Conclusion

This study clarifies the sterilization compatibility of 4PCLMA PolyHIPE scaffolds developed for clinical application by systematically comparing clinically relevant terminal sterilization methods with commonly used laboratory disinfection practices. The results demonstrate a clear distinction between disinfection and sterilization in three-dimensional porous constructs: while alcohol treatment and UV exposure were insufficient to ensure sterility throughout the interconnected pore network, terminal sterilization methods reliably eliminated microbial contamination without compromising scaffold morphology, mechanical performance, or cytocompatibility. These findings address a key practical barrier in the translational pathway of highly porous polymer scaffolds and support the progression toward preclinical evaluation under clinically relevant workflows.

The observed sterilization tolerance is closely associated with the thermoset network architecture of 4PCLMA PolyHIPE scaffolds, where cross-linking restricts polymer chain mobility and enhances structural stability during heat-based sterilization. In contrast, thermoplastic PCL-based scaffolds may be more susceptible to thermal deformation or crystallinity changes. These observations suggest that cross-link density and network structure represent important parameters governing sterilization compatibility in porous polymer systems, while also emphasizing that sterilization response remains material-specific and requires case-by-case evaluation.

While scaffold structure and biological performance were preserved immediately following sterilization, several aspects remain to be further explored. The present sterility assessment relied on qualitative growth-based detection, and future studies incorporating quantitative microbial recovery methods, such as CFU analysis, could provide additional validation of sterilization efficiency. Similarly, evaluation of additional cell markers would enable deeper insight into cell attachment and spreading behavior. Moreover, clinically established other sterilization routes not examined here, including irradiation-based methods and ethylene oxide sterilization, may induce distinct material responses that warrant dedicated investigation. Long-term degradation studies and *in vivo* evaluation will therefore be important to further assess material stability and translational performance.

Collectively, these findings highlight that sterilization compatibility should not be considered a late-stage validation step but rather an early-stage design criterion in the development of porous tissue engineering scaffolds intended for clinical translation.

## Supplementary Material



## Notes

XRD graph (Figure S1); original (uncolored) biological SEM images (Figure S2); summary of the related literature (Table S1) (PDF)
