# Supplementary material for: Effect of Sterilization and Disinfection Methods on the Physicochemical, Mechanical, and Biological Properties of Highly Porous Thermoset Polycaprolactone-Based Scaffolds
Source: ACS Omega. 2026 Mar 19;11(12):19880–93. doi: 10.1021/acsomega.6c00912 (PMC13044648; doi:10.1021/acsomega.6c00912)
Supplement: Supplementary file 1 [file ao6c00912_si_001.pdf]

Supporting Information

Effect of sterilisation and disinfection methods on the physicochemical, mechanical, and biological properties of highly porous thermoset polycaprolactone-based scaffolds

Helin Ozsel, Anıl Ceylan, Oğul Can Erdoğan, Betül Aldemir Dikici*

Izmir Institute of Technology, Department of Bioengineering, Urla, Izmir, 35430, Turkey

***Corresponding author:** betulaldemir@iyte.edu.tr

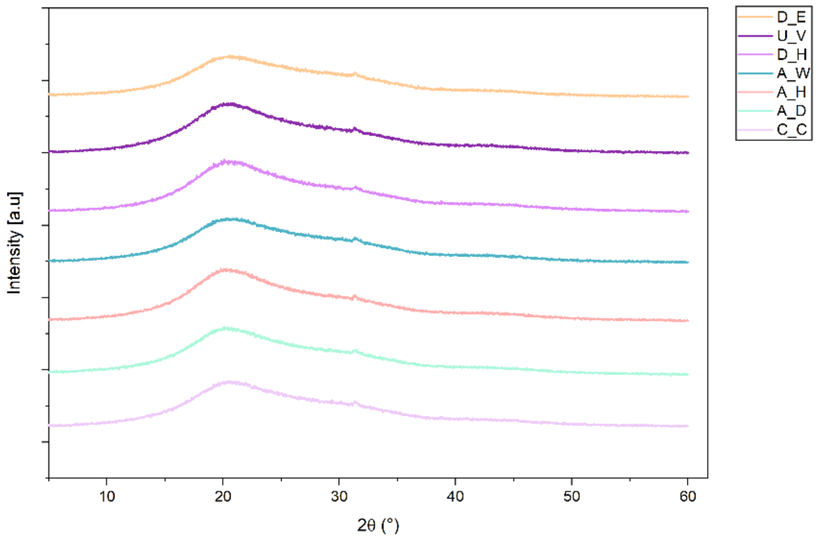

***Figure S1.** XRD patterns of treated 4PCLMA PolyHIPE scaffolds compared to the control.*

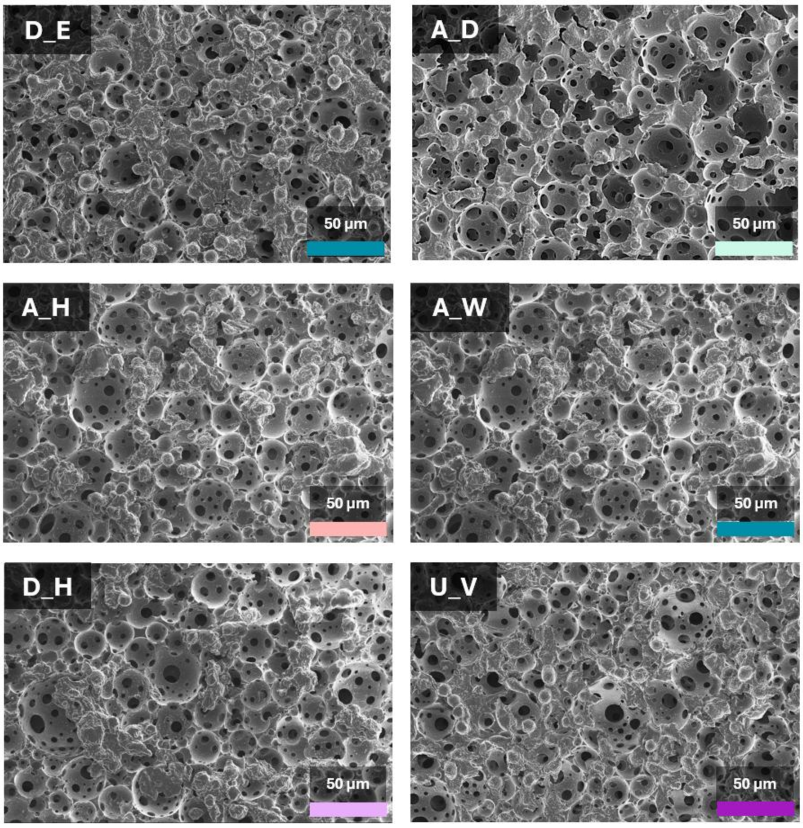

Figure S2. *Representative uncoloured biological SEM images showing L929 cell attachment and distribution on treated 4PCLMA PolyHIPE scaffolds.*

Table S1. *Summary of reported effects of different sterilisation and disinfection methods on characteristics of various scaffold materials.*

Scaffold material	Fabrication technique	Sterilisation / disinfection technique	Impact on material characteristics	Ref
**Polyurethane (PU)**	Solution blow spinning	Antimicrobial solution (AMS), UV irradiation, Gamma irradiation, E-beam, EtOH	- Porosity ↓ (EtOH and e-beam)<br>- Young modulus ↑ (AMS, EtOH, and e-beam)<br>- Metabolic activity↓ (UV group)	1
**Poly Lactic-co-Glycolic Acid (PLGA)**	Phase inversion/particulate leaching	70% EtOH, Gamma, RFGD plasma, Ethylene oxide	- 60% shrinkage (EtOH)<br>- 50% molecular weight ↓(gamma radiation)	2
**Polycaprolactone (PCL)**	Electrospinning	Ethylene oxide, Gamma irradiation, Peracetic acid (PAA), Hydrogen peroxide, EtOH, UV irradiation	- Loss of fibrous morphology (hydrogen peroxide)<br>- Highest and lowest proliferation rate in the Gamma irradiated and ETO treated groups, respectively	3
**Polyethylene Glycol (PEG), PEG-Chitosan, Multi-arm PEG**	Hydrogel/Mould casting	Gamma, 70% EtOH, Autoclave	- Shift in carboxyl and hydroxyl band shift in FTIR (gamma irradiation)	4
**Polyvinylidenefluoride (PVDF)**	Electrospinning	Autoclave, Ethylene oxide, Gamma, Hydrogen peroxide, EtOH	- Most and least fibre diameter changes obtained in the ETO treatment and gamma irradiation treatment groups, respectively<br>- Low cell proliferation (hydrogen peroxide)	5
**Polylactic acid (PLA)**	Additive manufacturing	Ultraviolet C (254nm) irradiation, Ultraviolet B+C (310nm) irradiation	- Difference in the yield displacement in all groups<br>- Difference in fracture displacement in ultraviolet B+C irradiation	6
**Decellularised pericardial**	Decellularisation	Low-energy electron irradiation	- Higher cell number and cell activity in the low-energy electron irradiated group compared to the non-sterilised group	7
**Silk Fibroin**	Salt leaching	Aseptic preparation, Dry, humid, and wet autoclave, Dry heat, Ethylene oxide, 70% EtOH, Antibiotic-antimyotic	- Maximum stress↓ (wet autoclave and aseptic)	8
**PCL**	Supercritical CO_2 foaming	Supercritical CO_2 extraction	- No changes in bioactivity, drug release profile, and morphology by the sterilisation method	9
**PCL/Beta-Tricalcium Phosphate (PCL/β-TCP)**	3D printing	Electron Beam	-14% ↑in stiffness and mechanical strength<br>-25% ↑in degradation rate	10
**Poly(ε-caprolactone-co-p-dioxanone) (PCLDX)**	Electrospinning	Ethylene oxide, Gamma	-Significant ↓ in molar mass and ↑of dales and hills in the morphology due to gamma irradiation	11
**Brain tissue**	Decellularisation	Supercritical CO_2, UV irradiation, Ethylene oxide	- ↓ cell viability due to ethylene oxide compared to UV irradiation and supercritical CO_2	12
**Sericin/polyvinyl alcohol/glycerin (Ser/PVA/Gly)**	Freeze-drying	Ethylene oxide, Gamma, 70% EtOH	- Shrinkage of scaffold and ↓ average pore size (EtOH)<br>- ↑ swelling degree (gamma irradiation)<br>- ↑ flexibility in Gamma irradiated and 70% EtOH treated scaffolds compared to control and ethylene oxide treated scaffolds	13
**Calcium phosphate, Bacterial cellulose**	Freeze-drying	Autoclave, UV irradiation, Microwave	- Slight darkening in colour in scaffolds sterilised by autoclave and microwave	14
**Chitosan (CS), Hyaluronic Acid (HA) and CS/HA**	Freeze-drying	UV irradiation, Gamma	- ↑ swelling equilibrium (gamma irradiation) (CS)<br>- ↓swelling equilibrium (UV-irradiation) (CS)	15
**Polyurethane (DegraPol)**	Electrospinning	UV irradiation	- Attenuation of the growth factor release kinetics	16
**PLGA, Poly(L-lactide-co-e-caprolactone) (P(LLA-CL))**	Electrospinning	UV irradiation	- ↑ degradation rate<br>- ↓ ultimate strength<br>- ↓ molecular weight	17
**Alginate**	3D printing	Autoclave, 70% EtOH, UV irradiation	- ↓in molecular weight, dynamic viscosity and polydispersity index (autoclave)<br>- Highest cell activity (autoclave) due to ↑ permeability by ↓ the molecular weight	18
**Collagen**	Freeze-drying	Ethylene oxide, Beta irradiation, Gamma, 70%, EtOH	- Increased degradation rate in irradiated scaffolds	19
**Collagen**	Freeze-drying	Ethylene oxide, Gamma	- Decrease of resistance against enzymatic degradation in gamma-irradiated scaffolds<br>- Reduction of surface area after cell inoculation in gamma-irradiated scaffolds	20
**PLGA, PLA, Collagen**	Commercial GTR Membranes *(PLGA (Biomesh), PLA(Epi-Guide), Collagen (BioMend and Collagen AT))*	Gamma, Ethylene oxide	- Impaired bone formation in ethylene oxide-treated scaffolds compared to gamma irradiation<br>- ↑resorption (gamma irradiation) compared to ethylene oxide treatment	21
**Poly(L-lactide-co-caprolactone) (PLCL)**	Commercial Sheet (Proxy Biomedical)	Electron beam, glow discharge, Ethylene oxide	- ↑ALP activity (ethylene oxide)<br>- ↓ contact angle ↑ surface free energy (glow discharge)	22
**Gelatin Methacryloyl (GelMA)**	Hydrogel/Mould casting	Autoclave, Ethylene oxide, Gamma	- ↓ stiffness (autoclave and ethylene oxide)<br>- ↑ stiffness, ↓ pore size and degradation rate (gamma irradiation)<br>- ↓ fibroblast viability (ethylene oxide)<br>- ↓ printability ↓ability of GelMA to undergo sol-gel transition (gamma irradiation)	23

References

(1) Łopianiak, I.; Butruk-Raszeja, B. A. Evaluation of Sterilization/Disinfection Methods of Fibrous Polyurethane Scaffolds Designed for Tissue Engineering Applications. *International Journal of Molecular Sciences 2020, Vol. 21, Page 8092* **2020**, *21* (21), 8092. https://doi.org/10.3390/IJMS21218092.

(2) Holy, C. E.; Cheng, C.; Davies, J. E.; Shoichet, M. S. Optimizing the Sterilization of PLGA Scaffolds for Use in Tissue Engineering. *Biomaterials* **2000**, *22* (1), 25–31. https://doi.org/10.1016/S0142-9612(00)00136-8.

(3) Horakova, J.; Klicova, M.; Erben, J.; Klapstova, A.; Novotny, V.; Behalek, L.; Chvojka, J. Impact of Various Sterilization and Disinfection Techniques on Electrospun Poly-ε-Caprolactone. *ACS Omega* **2020**, *5* (15), 8885–8892. https://doi.org/10.1021/ACSOMEGA.0C00503.

(4) Escudero-Castellanos, A.; Ocampo-García, B. E.; Domínguez-García, M. V.; Flores-Estrada, J.; Flores-Merino, M. V. Hydrogels Based on Poly(Ethylene Glycol) as Scaffolds for Tissue Engineering Application: Biocompatibility Assessment and Effect of the Sterilization Process. *Journal of Materials Science: Materials in Medicine 2016 27:12* **2016**, *27* (12), 176-. https://doi.org/10.1007/S10856-016-5793-3.

(5) Jirkovec, R.; Klápšťová, A.; Chvojka, J.; Horáková, J. INFLUENCE OF STERILIZATION METHODS ON NANOFIBROUS LAYERS MADE FROM PVDF. *NANOCON Conference Proceedings - International Conference on Nanomaterials* **2024**, 313–318. https://doi.org/10.37904/NANOCON.2023.4800.

(6) Aboamer, M. A.; Alsuayri, A. S.; Alassaf, A.; Alqahtani, T. M.; Alresheedi, B. A.; Saijari, G. N.; Osman, E. A.; Mohamed, N. A. R. Hybrid Radiant Disinfection: Exploring UVC and UVB Sterilization Impact on the Mechanical Characteristics of PLA Materials. *Polymers 2023, Vol. 15, Page 4658* **2023**, *15* (24), 4658. https://doi.org/10.3390/POLYM15244658.

(7) Walker, S.; Schönfelder, J.; Tugtekin, S. M.; Wetzel, C.; Hacker, M. C.; Schulz-Siegmund, M. Stabilization and Sterilization of Pericardial Scaffolds by Ultraviolet and Low-Energy Electron Irradiation. *https://home.liebertpub.com/tec* **2018**, *24* (12), 717–729. https://doi.org/10.1089/TEN.TEC.2018.0285.

(8) Hofmann, S.; Stok, K. S.; Kohler, T.; Meinel, A. J.; Müller, R. Effect of Sterilization on Structural and Material Properties of 3-D Silk Fibroin Scaffolds. *Acta Biomater.* **2014**, *10* (1), 308–317. https://doi.org/10.1016/J.ACTBIO.2013.08.035.

(9) Santos-Rosales, V.; Magariños, B.; Alvarez-Lorenzo, C.; García-González, C. A. Combined Sterilization and Fabrication of Drug-Loaded Scaffolds Using Supercritical CO2 Technology. *Int. J. Pharm.* **2022**, *612*, 121362. https://doi.org/10.1016/J.IJPHARM.2021.121362.

(10) Bruyas, A.; Moeinzadeh, S.; Kim, S.; Lowenberg, D. W.; Yang, Y. P. Effect of Electron Beam Sterilization on Three-Dimensional-Printed Polycaprolactone/Beta-Tricalcium Phosphate Scaffolds for Bone Tissue Engineering. *Tissue Eng. Part A* **2019**, *25* (3–4), 248–256. https://doi.org/10.1089/TEN.TEA.2018.0130.

(11) Morales López, Á.; Appaiah, A.; Berglund, J.; Marteleur, K.; Ajalloueian, F.; Finne-Wistrand, A. Effect of Ethylene Oxide and Gamma Sterilization on Surface Texture of Films and Electrospun Poly(ε-Caprolactone-Co-p-Dioxanone) (PCLDX) Scaffolds. *Polym. Test.* **2024**, *139*, 108567. https://doi.org/10.1016/J.POLYMERTESTING.2024.108567.

(12) Yaldiz, B.; Saglam-Metiner, P.; Cam, S. B.; Korkusuz, P.; Yesil-Celiktas, O. Effect of Sterilization Methods on the Mechanical Stability and Extracellular Matrix Constituents of Decellularized Brain Tissues. *J. Supercrit. Fluids* **2021**, *175*, 105299. https://doi.org/10.1016/J.SUPFLU.2021.105299.

(13) Siritientong, T.; Srichana, T.; Aramwit, P. The Effect of Sterilization Methods on the Physical Properties of Silk Sericin Scaffolds. *AAPS PharmSciTech* **2011**, *12* (2), 771–781. https://doi.org/10.1208/s12249-011-9641-y.

(14) Francisco, E. M.; De Oliveira Zoccolotti, J.; Tiomnova, O. T.; Tolaba, A. G.; Rodriguez Chanfrau, J. E.; Habib Jorge, J.; Basmaji, P.; Guastaldi, A. C. Sterilization of Scaffolds of Calcium Phosphates and Bacterial Cellulose for Their Use in Tissue Regeneration. **2021**, *11* (3), 10089–10098. https://doi.org/10.33263/BRIAC113.1008910098.

(15) Miranda, D. G.; Malmonge, S. M.; Campos, D. M.; Attik, N. G.; Grosgogeat, B.; Gritsch, K. A Chitosan-hyaluronic Acid Hydrogel Scaffold for Periodontal Tissue Engineering. *J. Biomed. Mater. Res. B Appl. Biomater.* **2016**, *104* (8), 1691–1702. https://doi.org/10.1002/jbm.b.33516.

(16) Evrova, O.; Kellenberger, D.; Scalera, C.; Calcagni, M.; Giovanoli, P.; Vogel, V.; Buschmann, J. Impact of UV Sterilization and Short Term Storage on the in Vitro Release Kinetics and Bioactivity of Biomolecules from Electrospun Scaffolds. *Scientific Reports 2019 9:1* **2019**, *9* (1), 15117-. https://doi.org/10.1038/s41598-019-51513-1.

(17) Yixiang, D.; Yong, T.; Liao, S.; Chan, C. K.; Ramakrishna, S. Degradation of Electrospun Nanofiber Scaffold by Short Wave Length Ultraviolet Radiation Treatment and Its Potential Applications in Tissue Engineering. *Tissue Eng. Part A* **2008**, *14* (8), 1321–1329. https://doi.org/10.1089/TEN.TEA.2007.0395.

(18) Chansoria, P.; Narayanan, L. K.; Wood, M.; Alvarado, C.; Lin, A.; Shirwaiker, R. A. Effects of Autoclaving, EtOH, and UV Sterilization on the Chemical, Mechanical, Printability, and Biocompatibility Characteristics of Alginate. *ACS Biomater. Sci. Eng.* **2020**, *6* (9), 5191–5201. https://doi.org/10.1021/ACSBIOMATERIALS.0C00806.

(19) *The Effect of Ethylene Oxide Sterilisation, Beta Irradiation and Gamma Irradiation on Collagen Fibril-Based Scaffolds*. https://www.researchgate.net/publication/241885605_The_Effect_of_Ethylene_Oxide_Sterilisation_Beta_Irradiation_and_Gamma_Irradiation_on_Collagen_Fibril-Based_Scaffolds (accessed 2026-01-05).

(20) Noah, E. M.; Chen, J.; Jiao, X.; Heschel, I.; Pallua, N. Impact of Sterilization on the Porous Design and Cell Behavior in Collagen Sponges Prepared for Tissue Engineering. *Biomaterials* **2002**, *23* (14), 2855–2861. https://doi.org/10.1016/S0142-9612(01)00412-4.

(21) Türker, N. S.; Özer, A. Y.; Kutlu, B.; Nohutcu, R.; Sungur, A.; Bilgili, H.; Ekizoglu, M.; Özalp, M. The Effect of Gamma Radiation Sterilization on Dental Biomaterials. *Tissue Engineering and Regenerative Medicine 2014 11:5* **2014**, *11* (5), 341–349. https://doi.org/10.1007/S13770-014-0016-9.

(22) Kroeze, R. J.; Helder, M. N.; Roos, W. H.; Wuite, G. J. L.; Bank, R. A.; Smit, T. H. The Effect of Ethylene Oxide, Glow Discharge and Electron Beam on the Surface Characteristics of Poly(l-Lactide-Co-Caprolactone) and the Corresponding Cellular Response of Adipose Stem Cells. *Acta Biomater.* **2010**, *6* (6), 2060–2065. https://doi.org/10.1016/J.ACTBIO.2009.11.022.

(23) Rizwan, M.; Chan, S. W.; Comeau, P. A.; Willett, T. L.; Yim, E. K. F. Effect of Sterilization Treatment on Mechanical Properties, Biodegradation, Bioactivity and Printability of GelMA Hydrogels. *Biomedical Materials* **2020**, *15* (6), 065017. https://doi.org/10.1088/1748-605X/ABA40C.
